# Protective effect of predator species richness on human hantavirus infection incidence

**DOI:** 10.1038/s41598-020-78765-6

**Published:** 2020-12-10

**Authors:** Kyung-Duk Min, Ho Kim, Seung-sik Hwang, Seongbeom Cho, Maria Cristina Schneider, Jusun Hwang, Sung-il Cho

**Affiliations:** 1 Institute of Health and Environment, Graduate School of Public Health, Seoul National University, Seoul, South Korea; 2 Department of Public Health Science, Graduate School of Public Health, Seoul National University, Seoul, South Korea; 3 College of Veterinary Medicine and Research Institute for Veterinary Science, Seoul National University, Seoul, South Korea; 4 Department of International Health, School of Nursing and Health Sciences, Georgetown University, Washington, DC USA; 5 Institute of Collective Health Studies, Federal University of Rio de Janeiro, Rio de Janeiro, Brazil; 6 Wildlife Conservation Society, New York, USA

**Keywords:** Risk factors, Infectious diseases

## Abstract

Are predators of rodents beneficial for public health? This question focuses on whether predators regulate the spillover transmission of rodent-borne diseases. No clear answer has emerged because of the complex linkages across multiple trophic levels and the lack of accessible data. Although previous empirical findings have suggested ecological mechanisms, such as resource partitioning, which implies protective effects from predator species richness, epidemiological evidence is needed to bolster these arguments. Thus, we investigated the association between predator species richness and incidence of rodent-borne haemorrhagic fever with renal syndrome in the human population using district-level longitudinal data of 13 years for South Korea. With the exception of districts with low species richness, we found a significant negative association between the incidence of haemorrhagic fever with renal syndrome and the species richness of both avian and mammalian predators; the trends for both predator types were similar. Thus, biodiversity conservation may benefit public health.

## Introduction

The relationship between wildlife diversity and the risk of zoonotic disease transmission to humans (spillover transmission) has become increasingly investigated given global concerns about both biodiversity loss and the increasing threats to human health posed by wildlife-associated infectious diseases. Most studies highlighted the protective effects of host diversity. For example, a higher diversity of reservoir rodents was associated with a lower prevalence of tick-borne Lyme disease among the rodents^1^, possibly due to regulatory pressure exerted through mechanisms such as reduced abundance and/or activity of high-competency reservoirs^2^. This phenomenon is termed the “dilution effect”. This hypothesis has been disputed, and some results are inconclusive^3^ or even contrary to this suggestion^4^, implying that any negative association between diversity and the spillover risk depends on specific interactions among species, i.e. on the presence and/or abundance of certain species in a community. On the other hand, accumulating evidence from analytical modelling^5,6^, empirical findings^7,8^, and experimental studies^9,10^ strongly support the dilution effect. However, this remains controversial, and the field is evolving.

Such an approach to correlating public health with host biodiversity has been extended to investigations of the beneficial effects of predators on public health. Especially, previous studies focused on the effects of predation pressure on the spillover transmission of rodent-borne diseases from reservoir hosts to human populations. O’Bryan et al.^11^ reviewed previous studies that reported the protective health effects of predators. For example, reduced density of vultures was associated with increased abundance of feral dogs, which could lead to frequent dog bites and higher rabies incidence in the human population^12^. In addition, the presence of red fox was associated with a lower burden of Lyme disease^13^. Furthermore, studies on the effects of predator species richness, which indicated the number of species rather than the presence of a single predator species, have also been conducted. These studies suggested that higher predator species richness was associated with lower disease prevalences in rodent and vector populations^14,15^, explained by the enhanced regulatory pressure imposed on rodents when the predator species richness increases. Earlier empirical studies suggested that various predators can increase the predation pressure, potentially reducing the abundance^16^ and/or activities^17^ of prey individuals. One possible underlying mechanism is resource partitioning; competing predators tend to use different niches to increase their chance of coexistence, consequently decreasing the prey density^16^.

However, such findings of studies of community ecology or disease ecology should be complemented by epidemiological studies using direct measures of human disease risk (e.g. incidence rate of human disease) to bolster the suggestion that public health benefits from predator richness. For example, the effects of predators would likely be limited to highly intact regions where the contact rate between humans and reservoir is usually low. In this case, the effects of predator species richness could reduce the prevalence of infection among reservoirs, but the health benefit to the human population may not be significant. Moreover, epidemiological studies using direct measures can provide information about the size of the association, which would be useful for health authorities.

In this regard, we conducted a national level observational study to assess the preventive effects of predator species richness on human disease risk, specifically, on human incidence of rodent-borne haemorrhagic fever with renal syndrome (HFRS) in South Korea. The study of South Korea provided advantageous opportunities to investigate the association due to the endemicity of HFRS and the richness of available data. South Korea has an annual incidence of HFRS of 300–600 cases, and these are distributed all over the country (Supplementary Material 1). In addition, the National Institute of Ecology (NIE) conducted a national ecosystem survey on wildlife, which enabled us to estimate local-level species richness of predators^18^. Various datasets from the Korean Statistical Information Service (KOSIS)^19^ and the Korea Meteorological Administration (KMA)^20^ provided us with covariates, such as sociodemographic, meteorological, and geographic factors, which were needed to adjust the association between HFRS incidence and predator richness. Details of the data acquisition and pre-processing are described in the Methods section of this paper and in Supplementary Materials 2 and 3. When assessing predator species richness, we included both mammalian and avian species, and the association of each class was examined separately.

## Results

### General characteristics of the study area

Descriptive analyses of variables including demographic, socioeconomic, meteorological, and geographical factors of the 250 districts in South Korea are presented in Table 1, with average values between 2006 and 2018. Categorizing the districts by median of annual mean HFRS incidence (7.87 cases per 100,000), we overviewed differences between districts with higher HFRS cases and districts with lower HFRS cases. The districts with fewer HFRS cases tended to have urban-like characteristics. For example, predator species richness, the species richness of reservoirs, agricultural land use, and farmer population were lower than those of districts with higher HFRS cases. In addition, population density and proportion of urban land use were higher in the districts with lower HFRS. There was no significant difference in annual precipitation or urbanized area between the two groups of districts.

**Table 1:** Descriptive analysis of variables included in this study according to districts with higher versus lower HFRS values.

Variable	Mean (± standard deviation)		*P-*value^b^
	Lower HFRS^a^ (≤ median, N = 125)	Higher HFRS^a^ (> median, N = 125)	
HFRS cases per 100,000 (annual mean)	3.21 ± 1.9	30.57 ± 23.3	< 0.001
Predator species richness	4.44 ± 4.1	10.11 ± 3.0	< 0.001
Reservoir species richness	1.34 ± 1.4	3.42 ± 1.5	< 0.001
Deforestation (2006–2018, sum, km^2^)	2.04 ± 5.2	10.85 ± 11.1	< 0.001
Population density (10^3^ per km^2^)	7.69 ± 7.0	0.36 ± 0.8	< 0.001
Number of farmer population (10^3^)	5.84 ± 9.0	17.07 ± 8.1	< 0.001
Budget dependency (%)	36.77 ± 15.6	22.15 ± 12.9	< 0.001
Average mean temperature (°C)	12.98 ± 1.1	12.62 ± 0.9	0.006
Annual precipitation (mm)	1310.24 ± 123.0	1287.68 ± 124.7	0.151
Relative humidity (%)	66.65 ± 2.3	68.67 ± 2.8	< 0.001
Agriculture (paddy, m^2^)	915.04 ± 1799.2	6736.35 ± 5314.4	< 0.001
Urban area (km^2^)	70.99 ± 79.7	65.24 ± 73.9	0.555
Forested area (km^2^)	74.97 ± 154.7	283.43 ± 269.3	< 0.001
Elevation (mean, m)	125.46 ± 122.1	215.28 ± 160.9	< 0.001
Area (km^2^)	171.52 ± 262.4	633.46 ± 323.9	< 0.001

The descriptive analyses were conducted using data for 13 years (2006–2018) for 250 districts in South Korea.

^a^Annual cases of haemorrhagic fever with renal syndrome per 100,000 population in each district (median = 7.87).

^b^*P* values from *t*-tests.

### Association between predator species richness and human HFRS incidence

**Figure 1:**
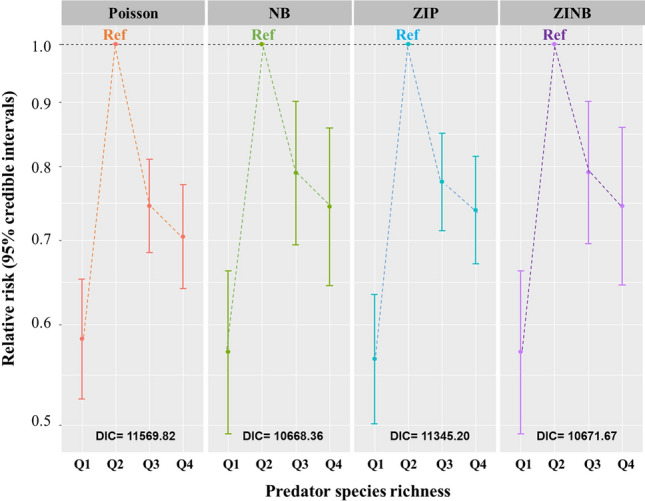
Association between the incidence of haemorrhagic fever with renal syndrome and predator species richness variables. Four models were employed, including the Poisson, negative binomial (NB), zero-inflated Poisson (ZIP), and zero-inflated negative binomial (ZINB) models, and the second quartile served as the reference. The NB model was selected as the best fit model due to its lowest DIC. The covariates included reservoir species richness, extent of deforestation, budget dependency, annual mean temperature, annual precipitation, relative humidity, agricultural area, urban area, and elevation (Q1–4 indicate 0–3, 4–8, 9–11, and 12–17, respectively).

To examine the linear associations between the incidence of HFRS and predator species richness with adjustment of spatial and temporal autocorrelations, Fig. 1 shows the results of spatiotemporal regression models. From among the four models, the Poisson, negative binomial (NB), zero-inflated Poisson (ZIP), and zero-inflated negative binomial (ZINB) models, the NB model, which showed the lowest deviance information criterion (DIC), was selected as the final model. The first (lowest richness), third, and fourth (highest richness) quartiles were associated with significantly lower risks, when using the second quartile as the reference; the association was thus reverse U-shaped. The relative risks (RRs) (95% confidence interval; CI) for the first, third, and fourth quartiles were 0.571 (0.492–0.662), 0.791 (0.694–0.901), and 0.744 (0.644–0.858), respectively.

**Figure 2:**
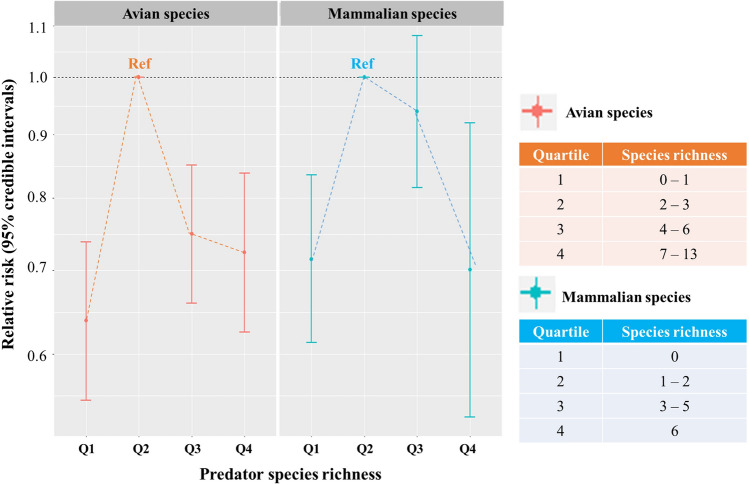
Association between the incidence of haemorrhagic fever with renal syndrome and predator species richness according to the predator class. Negative binomial models were employed, and the second quartile served as the reference. The covariates included reservoir species richness, extent of deforestation, budget dependency, annual mean temperature, annual precipitation, relative humidity, agricultural area, urban area, and elevation.

Using the NB model, we also examined the association between HFRS incidence and the species richness of each avian and mammalian predator (Fig. 2). For avian predators, the first (the lowest richness), third, and fourth (the highest richness) quartiles showed significantly lower risks relative to the second quartile, revealing a reverse U-shaped association (RR (95% CI): 0.638 (0.551–0.738), 0.749 (0.659–0.851), and 0.724 (0.625–0.838) for the first, third, and fourth quartiles, respectively). On the other hand, for mammalian predators, the first (lowest richness) and fourth (highest richness) quartiles exhibited significantly lower risks relative to the second quartile. Again, the association was reverse U-shaped (RR (95% CI): 0.715 (0.613–0.835) and 0.701 (0.534–0.920) for the first and fourth quartiles, respectively).

## Discussion

We examined the association between predator species richness and incidence of human HFRS in South Korea, hypothesizing that higher species richness of predators may suppress activities of reservoir rodents and thus reduce the risk of spillover transmission to the human population. We found that the first (the lowest), third, and fourth (the highest) quartiles of predator richness were associated with a significantly lower incidence of HFRS than the second quartile of predator richness, representing a reverse U-shaped association.

It was an unexpected finding that the lowest level of predator richness was associated with a lower HFRS incidence than the mid-level richness, irrespective of the model used or the class of predator species. A possible explanation is that there could be a relatively low abundance of reservoirs in the regions of lowest predator richness. Because we could not include abundance variables in this study, follow-up studies employing these variables may provide a more evident explanation. Except for the lowest level of predator species richness, the association between predator species richness and HFRS incidence was significantly negative which supported our hypothesis, the regulatory effect of predator species richness. While the trend was maintained when the predator species richness was separately included as avian and mammalian species, the difference of HFRS risk between districts with the second and third quartile of mammalian predator richness was not significant. It was possibly because the differences in the species richness was not sufficient to show the significance.

In terms of associations with covariates (Supplementary Material 4), the estimated higher risks in districts exhibiting greater deforestation, lower annual temperatures, and higher relative humidity were related to the ecology of the striped field mouse. As a generalist species, this mouse is resilient in deforested and disturbed areas and has even increased in abundance^21^. A previous study suggested that the risk of scrub typhus, of which the striped field mouse is a major reservoir, tended to increase in habitats with greater deforestation^22^. The positive association between humidity and the incidence of HFRS is consistent with the findings of a previous study in China^23^; increased rodent activity and numbers under high-moisture conditions explain this association. Rodents forage more intensively at lower temperatures because more calories are required to maintain their body temperature^24^. In addition, we found positive associations between reservoir species richness and the incidence of HFRS, contrary to the dilution effect hypothesis. As we did not include the abundance of reservoir species, a higher species richness may indicate higher abundance in South Korea.

Compared to the findings from a previous ecological study, which showed a clear linear negative association between predator richness and hantavirus prevalence among reservoirs^14^, our results were more complex and the negative associations were distinct only out of districts with low species richness where reservoir abundance would be low. However, our results still support the important health benefit of predator richness because the diseases arising from spillover transmission are often prevalent in areas with high abundance of reservoirs. The estimated RRs were high enough to be of interest in the public health field, considering that the predator richness barely fluctuates and thus that the effect could be long-term and chronic.

The study had several limitations that should be considered. First, there are some caveats regarding the species richness variables that we used. Because we assumed that species richness was static across the study period, possible temporal changes could not be represented. However, considering that the variables were obtained from the third National Ecosystem Survey, which was a long-term study conducted between 2006 and 2013, the likelihood of drastic changes between 2014 and 2018 could be low. Another consideration is the method of assessing local species richness. We did not employ a modelling approach, such as species distribution modelling^25^. Instead, we used observational reports of species occurrence and added the numbers of observed species, assuming that the 8-year-long search efforts were sufficient for reliability. Different methods of calculating species richness would provide different results. Second, the level of public health interventions, such as vaccination or public campaigns, was not included. However, the interventions would not be confounders of the association between species richness and the incidence of HFRS because effects of the interventions on species richness are unlikely. Third, the effects of evenness of predators were not examined because abundance data for each predator species were not available. Although several field surveys have been conducted in South Korea, a systematic survey covering the entire country with an identical methodology is needed for additional studies. Fourth, the unit of this study, the district, would affect the results, as a different scale of study unit (e.g. 1 km^2^ grid) would produce a different sample size and values of explanatory variables (i.e. modifiable area unit problem^26^). In addition, the outcome variable, i.e. the incidence of HFRS, cannot differentiate subtype of the virus (i.e., there was no information on whether it was Hantaan virus or Seoul virus etc.) or reservoirs which transmitted the virus. The effect of predator species richness on the infection risk of the various hantavirus subtypes may differ. For example, the effect of predator species richness on the infection risk Seoul virus, which is prevalent in urban areas, may be limited because predator pressure is low in urban areas^27^. Further experimental field studies examining differences in predatory pressures among reservoir species are needed.

However, our results indicate that the implications of the One Health Approach are plausible; this approach seeks to integrate humans, animals, and the environment in a multidisciplinary manner^28^. For instance, preventing local extinctions of predators by developing conservation activities or implementing restoration projects for previously eliminated species can be recognized as public health activities previously exclusively considered purely environmental concerns. As thousands of plant and animal species face extinction, global efforts to reduce biodiversity loss are being revised, as the existing approaches are inadequate^29^. Our study highlights the importance of biodiversity and the possible implications of biodiversity for public health. In addition, as a pragmatic measure, predator species richness could be used to predict risk areas of spillover transmission. Further studies are highly recommended to generalize the effects of predators in different regions and for different diseases.

## Methods

### Data acquisition and preprocessing

To examine the study’s hypothesis, various types of data including HFRS incidence, species richness of wildlife, and other covariates (including sociodemographic, meteorological, and geographic factors) were acquired from various sources. The variables used and the covariate selection strategy are described in Supplementary Material 2. The numbers of HFRS cases were obtained from the infectious disease portal of the Korean Center for Disease Control and Prevention (KCDC)^30^. The KCDC provides the annual number of cases by sex and age group at the national level and also the total number of cases at the district level. The expected number of HFRS cases was calculated using the total number of cases by sex and age group and the population structure (i.e. sex and age) of each district, assuming same age and sex-specific incidence rates. Total population size, number of farmers, budget dependency, urban area, agricultural area, and total area for each district and each year were obtained from KOSIS^19^.

Global-level spatial data from the IUCN representing the geographic range of each mammalian species were available^31^, but these data are not suitable for studies in South Korea because South Korea is a relatively small country and the IUCN data may not capture local level differences. Consequently, species occurrence reports from the national ecosystem survey^18^ conducted by the NIE between 2006 and 2013 were employed. The NIE provided district-level occurrence data for every target species, including mammals, birds, reptiles, and amphibians. Among these, we included species in the categories of Accipitridae, Falconidae, Strigidae, and Carnivora as predator species to compute predator species richness. Reservoir species of HFRS were also included based on the previous literature (Supplementary Material 5).

Meteorological factors, including annual average temperature, annual average relative humidity, and annual precipitation, were obtained from the automatic synoptic observation system^20^ of the KMA, which has fully open access from the KMA website. The data were provided by observation site with the corresponding location information (i.e. coordinates). To acquire representative values of the meteorological factors for each district, we implemented ordinary kriging, a spatial interpolation method, for each year and each factor. The geoR package^32^ was used for the implementation with R v.3.5.1^33^.

As a proxy for land cover change, deforestation data from the Global Forest Change database^34^ were used. To estimate deforestation, we used both a forest cover variable, which showed the level of forest cover in the year 2000, and a tree-loss variable, which indicated deforestation events by date^34^. Both were obtained from raster-type data with a resolution of approximately 30 m. The forest cover variable, representing the probability of the presence of tree canopy, ranged from 0 to 100, and we used 50 as the threshold for presence of forest. The tree-loss variable ranged from 1 to 18, indicating the year of deforestation occurrence from 2001 to 2018. Combining the two variables, we computed both forest land use and deforestation level during the previous 3 years for each district and each year. The calculation process followed a previous study^22^.

Elevation data were obtained from Jarvis et al.^35^ who provided altitude data at 90 m resolution. The elevation variables were provided in raster format. We extracted the elevation values for each district and averaged them.

Some covariates were not included in the final analyses. A variable selection process was implemented to avoid multicollinearity (Supplementary Material 3). As suggested in previous studies, variables with a variance inflation factor > 10^36^ were excluded, as were variables exhibiting one-to-one correlation coefficients > 0.7^37^.

The Institutional Review Board (IRB) of Seoul National University waived the need for informed consent (IRB approval number: SNU IRB number: E1811/001-014). The study was approved by the IRB of Seoul National University. All work adhered to all relevant guidelines and regulations.

### Statistical analysis

Descriptive analyses were conducted to overview differences in the selected variables between districts with higher incidence of HFRS cases and districts with lower incidence from 2006 to 2018 based on the median value of annual incidence. Means and standard deviations were provided, and choropleth maps with decile values were drawn to help clarify the spatial distributions of the variables. (Supplementary Material 1).

The unit of analysis was the district-year, and the total number of units was 3250 (250 districts over 13 years). Four models, the Poisson, NB, ZIP, and ZINB models, were employed to examine the associations between predator species richness and the incidence of HFRS, with consideration of potential over-dispersion and “excessive zero” cases. The expected number of HFRS cases served as an offset for models accounting for variations in population size and structure among districts. Initially, we used generalized additive models to examine possible non-linear associations between predator species richness and HFRS incidence. As the associations were non-linear and reverse U-shaped (Supplementary Material 6), we categorized predator richness into quartiles to provide intuitive effect sizes (i.e. RRs) among categories. The goodness-of-fit of each of the four models (Poisson, NB, ZIP, and ZINB) was examined using the DIC^38^, and the model with the lowest DIC was selected. Then, we conducted additional analyses using that model for avian and mammalian predator species richness values separately. Considering the possibility of autocorrelations by both time (year) and space, hierarchical approaches were incorporated^39^. To derive Bayesian inferences, integrated nested Laplace approximation approaches^40^ were employed by INLA package^41^ in R v. 3.5.1^33^. The results of all models are presented as RRs with 95% CIs.

## Supplementary Information

Supplementary Information

## Data Availability

The data sets are available from the corresponding author on reasonable request.

## Code availability

All data and code used to generate the models are available from the corresponding author on reasonable request.
