# Supplementary material for: Protective effect of predator species richness on human hantavirus infection incidence
Source: Sci Rep. 2020 Dec 10;10:21744. doi: 10.1038/s41598-020-78765-6 (PMC7728771; doi:10.1038/s41598-020-78765-6)
Supplement: Supplementary file 1 — Supplementary Information [file 41598_2020_78765_MOESM1_ESM.docx]

**Title: Protective effect of predator species richness on human hantavirus infection incidence**

Kyung-Duk, Min^1^, Ho Kim^1,2^, Seung-sik Hwang^1,2^, Seongbeom Cho^3^, Maria Cristina Schneider^4,5^, Jusun Hwang^6^, Sung-il Cho^1,2^*

^1^ Institute of Health and Environment, Graduate School of Public Health, Seoul National University

^2^ Department of Public Health Science, Graduate School of Public Health, Seoul National University

^3^ College of Veterinary Medicine and Research Institute for Veterinary Science, Seoul National University

^4^ Department of International Health, School of Nursing and Health Sciences, Georgetown University

^5^ Institute of Collective Health Studies, Federal University of Rio De Janeiro

^6^ Wildlife Conservation Society

* Corresponding author: Sung-il Cho

Academic degree: MD, ScD

Address: Department of Public Health Science, Graduate School of Public Health, Seoul National University, 1 Gwanak-ro, Gwanak-gu, Seoul, 08826, Korea

E-mail: [persontime@hotmail.com](mailto:persontime@hotmail.com)

**Supplementary material 1 – Temporal trend of hemorrhagic fever with renal syndrome and geographic distribution of included variables**

**
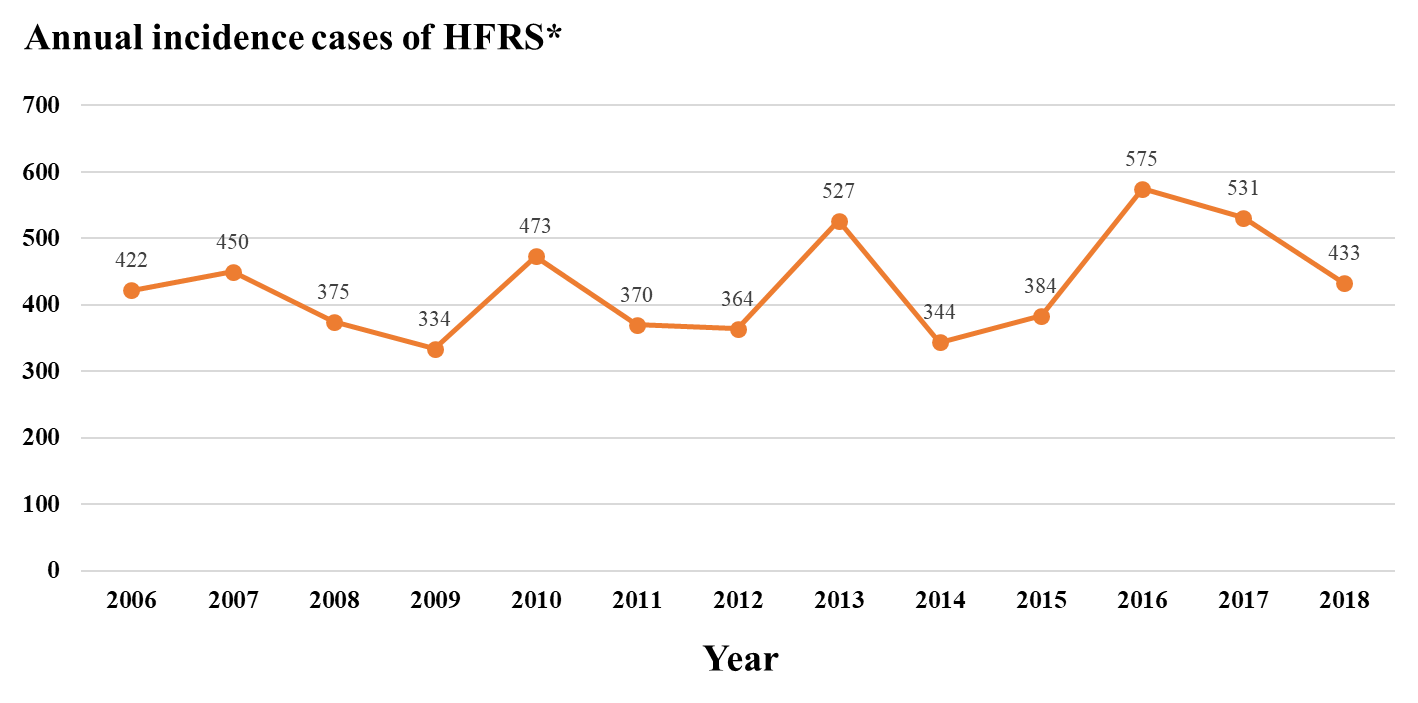
**

**Figure S1-1. Annual incidence cases of Hemorrhagic Fever with Renal Syndrome (HFRS) in South Korea from 2006 to 2018.**

**
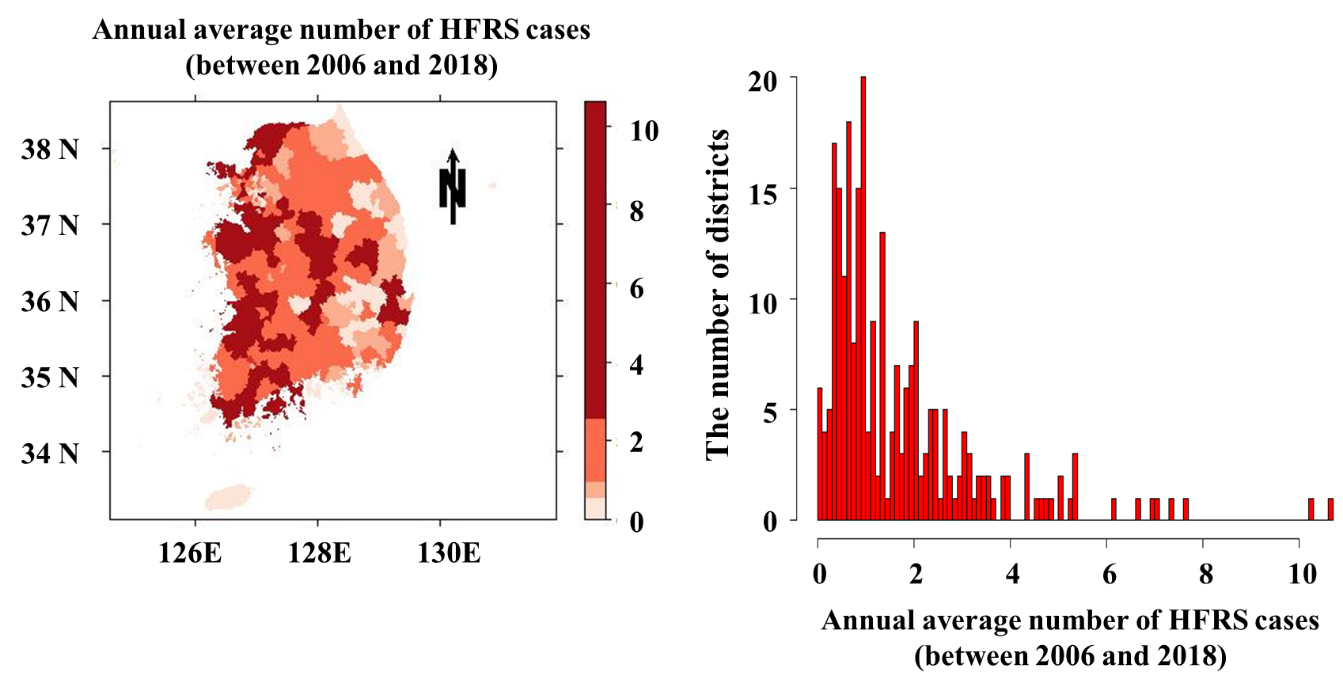
**

**Figure S1-2. Geographical distribution and histogram of district-level incidence cases of Hemorrhagic Fever with Renal Syndrome (HFRS) in South Korea**

**
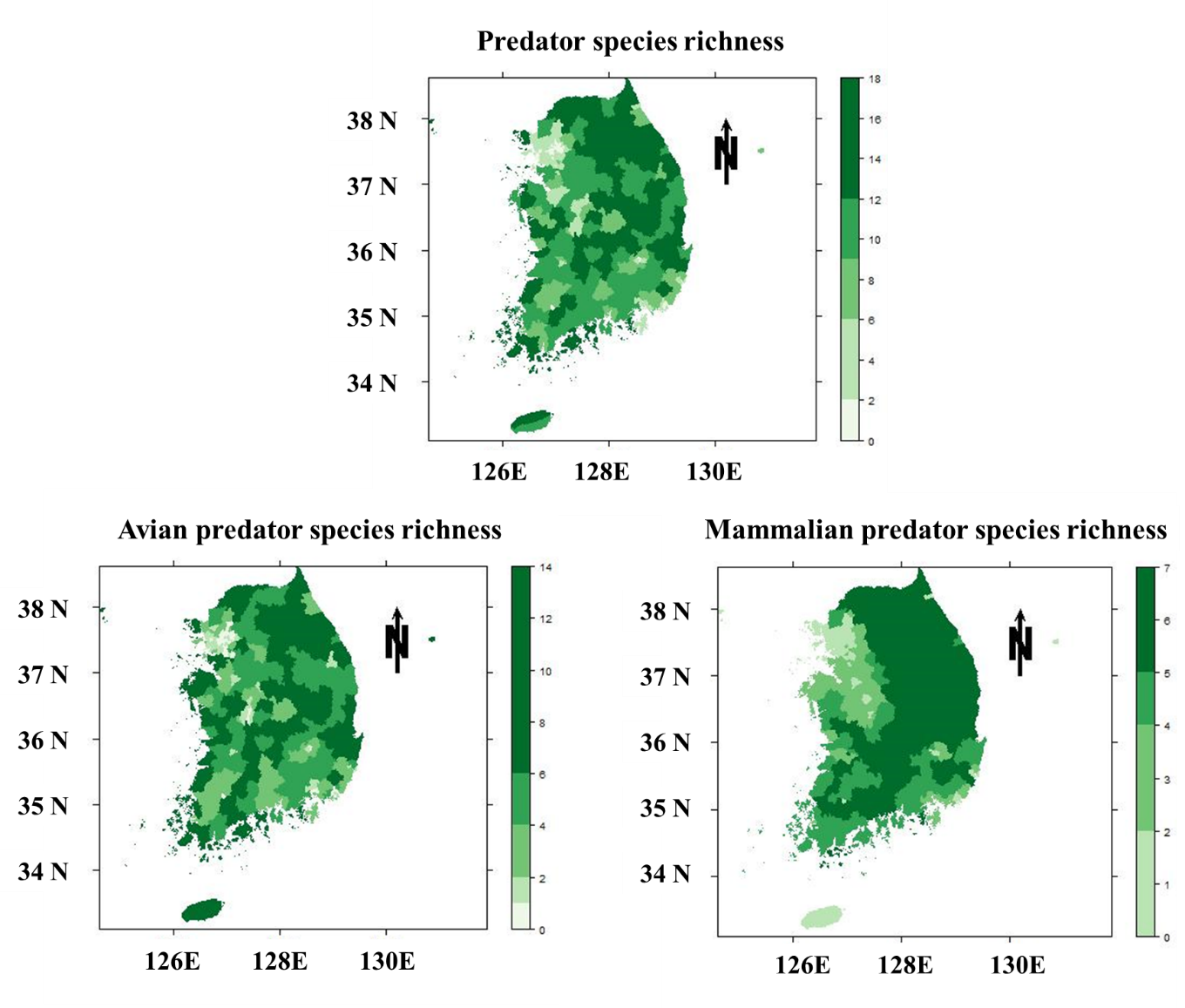
**

**Figure S1-3. Geographic distribution of predator species richness**

**
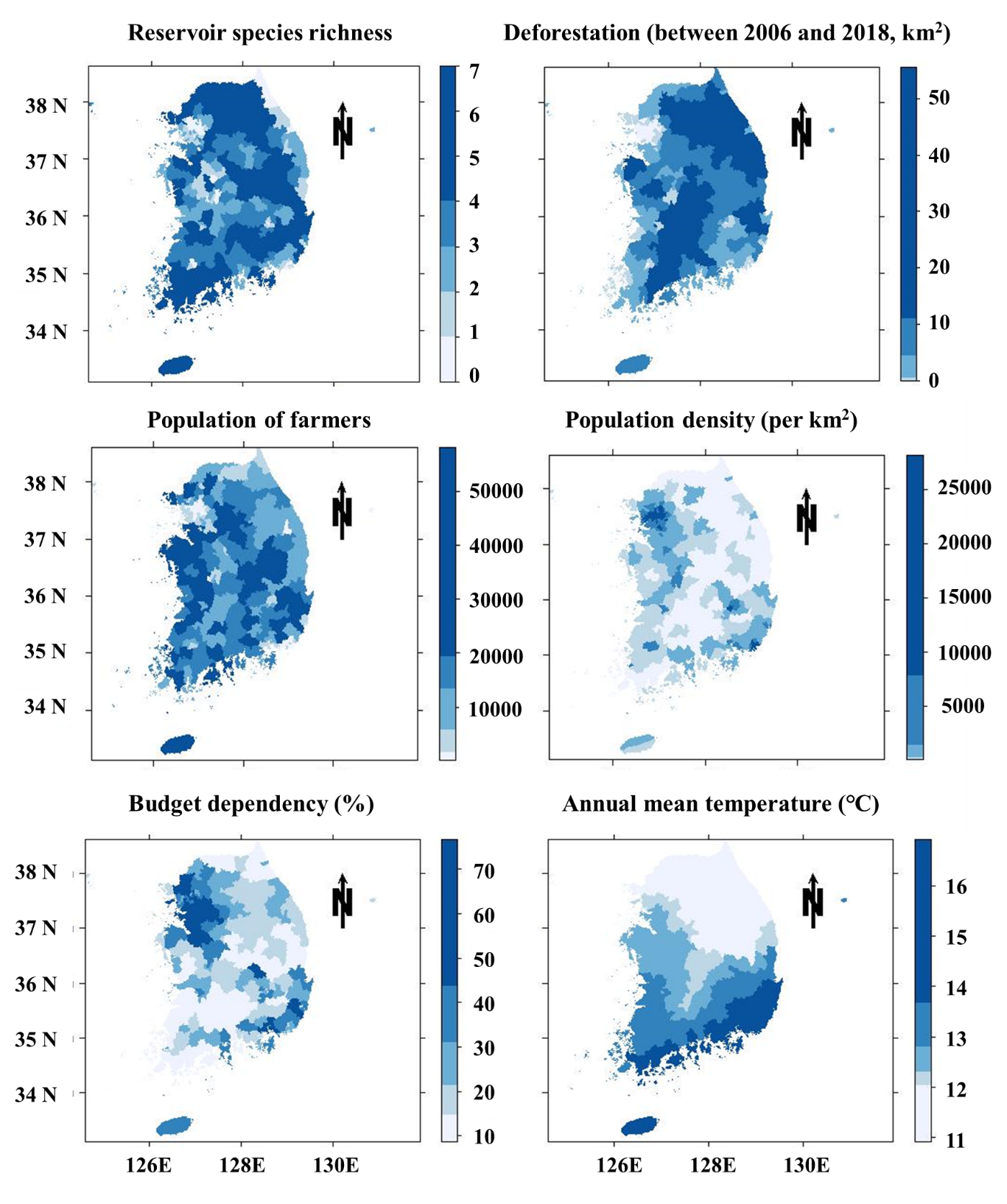
**

**Figure S1-4. Geographic distribution of covariate variables**

**
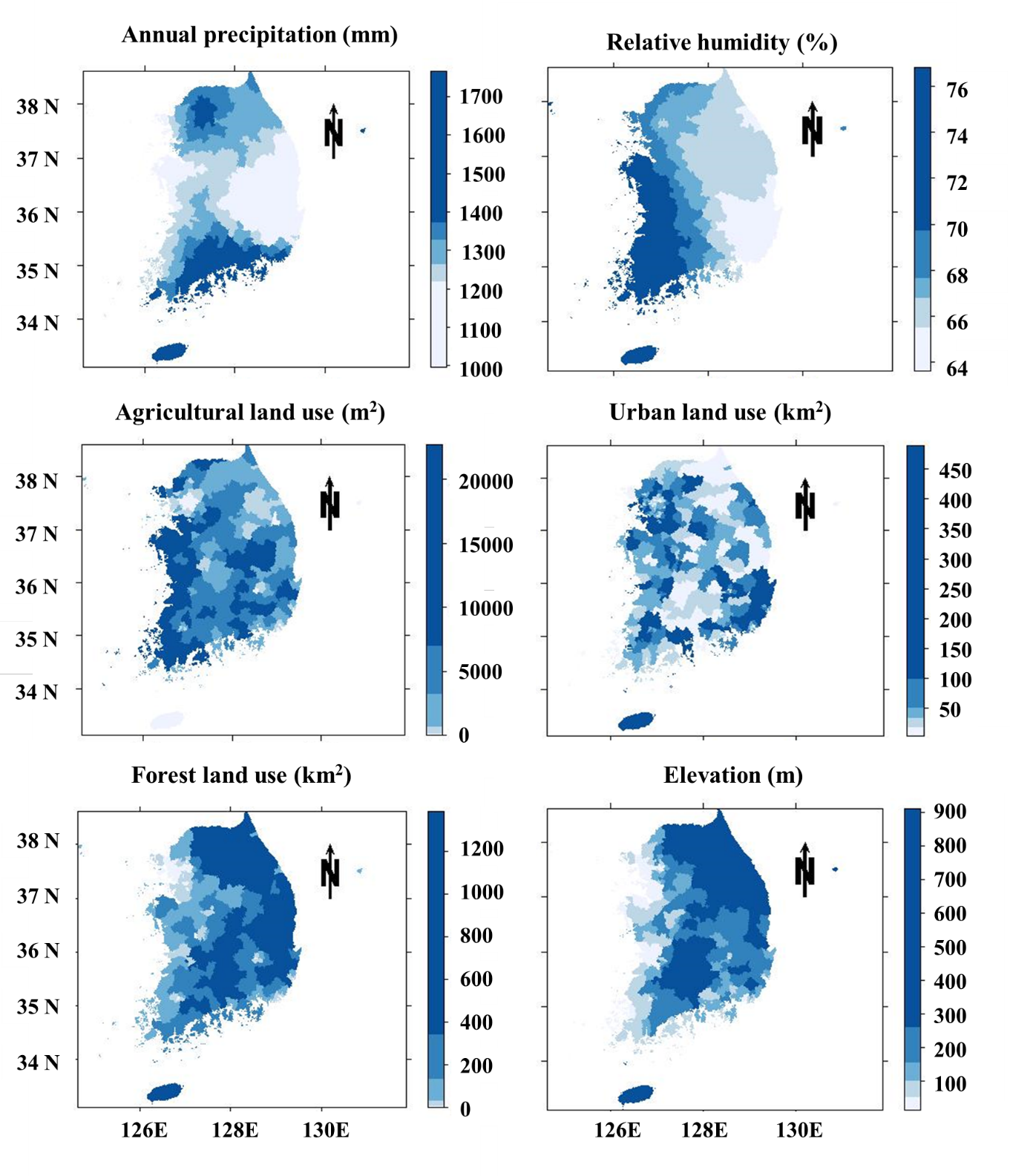
**

**Figure S1-4. Geographic distribution of covariate variables (cont.)**

**Supplementary material 2 – List of the covariates used in this study**

Table S2-1. List of acquired data.

| Variable | Unit | Time span | Used in the model |
| --- | --- | --- | --- |
| HFRS incidence | Case | 2006–2018 | Annual sum for each year |
| Predator species richness | NA (count) | 2006–2013 |  |
| Reservoir species richness | NA (count) | 2006–2013 |  |
| Extents of deforestation | km^2^ | 2006–2018 | Total for each district  (3-year cumulative) |
| Population density | 10^3^/km^2^ | 2006–2018 |  |
| Number of farmers | NA (count) | 2006–2018 |  |
| Budget dependency | % | 2006–2018 |  |
| Annual average temperature | ^°^C | 2006–2018 | Annual mean |
| Annual precipitation | mm | 2006–2018 | Annual sum |
| Relative humidity | % | 2006–2018 | Annual mean |
| Agricultural land use ^a^ | 100 m^2^ | 2006–2018 | Total size for each district |
| Urban land use | km^2^ | 2006–2018 | Total size for each district |
| Forest land use | km^2^ | 2006–2018 | Total size for each district |
| Elevation | m |  | Mean for each district |
| District area | km^2^ |  |  |

^a^: Agricultural land use: rice paddy.

Table S2-2. Data sources and methods used to pre-process the acquired data.

| Variable | Data source | Pre-processing method |
| --- | --- | --- |
| HFRS incidence | KCDC^1^ | - |
| Predator species richness | NIE^2^ | - |
| Reservoir species richness | NIE | - |
| Extent of deforestation | GFC^3^ | Extraction |
| Population density | KOSIS^4^ | - |
| Number of farmers | KOSIS | - |
| Budget dependency | KOSIS | - |
| Annual average temperature | KMA ASOS^5^ | Kriging, extraction |
| Annual precipitation | KMA ASOS | Kriging, extraction |
| Relative humidity | KMA ASOS | Kriging, extraction |
| Agricultural land use ^a^ | KOSIS | - |
| Urban land use | KOSIS | - |
| Forest land use | GFC | Extraction |
| Elevation | SRTM^6^ | Extraction |
| District area | KOSIS | - |

^a^: Agricultural land use: rice paddy.

*Note*: KCDC, Korea Centers for Disease Control; NIE, National Institute of Ecology; GFC, Global Forest Change; KOSIS, Korean Statistical Information Service; KMA ASOS, Korea Meteorological Agency Automated Surface Observing System; SRTM, Shuttle Radar Topography Mission

**Reference**

1 Park, S. & Cho, E. National Infectious Diseases Surveillance data of South Korea. Epidemiology and Health 36, e2014030, doi:10.4178/epih/e2014030 (2014).

2 Kim, C.-H., Kang, J.-H. & Kim, M. Status and development of national ecosystem survey in korea. Journal of environmental impact assessment 22, 725-738 (2013).

3 Hansen, M. C. et al. High-Resolution Global Maps of 21st-Century Forest Cover Change. Science 342, 850-853, doi:10.1126/science.1244693 (2013).

4 KOSIS. Korean Statistical Information Service. (2014).

5 Korea Meteorological Administration. Automatic Synoptic Observation System, <https://data.kma.go.kr/data/grnd/selectAsosRltmList.do?pgmNo=36> (2018).

6 Jarvis, A., H.I. Reuter, A. Nelson, E. Guevara, 2008, Hole-filled SRTM for the globe Version 4, available from the CGIAR-CSI SRTM 90m Database (http://srtm.csi.cgiar.org).
https://cgiarcsi.community/data/srtm-90m-digital-elevation-database-v4-1/

We selected 13 covariates (reservoir species richness, extent of deforestation, population density, number of farmers, budget dependency, annual average temperature, annual precipitation, relative humidity, agricultural land use, urban land use, forest land use, elevation, and spatial extent) when adjusting the association between predator species richness and human haemorrhagic fever with renal syndrome (HFRS). The selected covariates might confound the association. In other words, the selected covariates that could affect both predator species richness and spillover transmission of HFRS, or (at least) the spillover transmission risk, to reduce random error in the models (Table S2-3).

Table S2-3. The rationales used to select covariates.

| Covariates | Possible associations with | |
| --- | --- | --- |
|  | Predator species richness | Spillover transmission |
| Reservoir species richness | A higher reservoir species richness might increase the predator species richness. | A higher reservoir species richness with lower total abundance might decrease spillover transmission (a dilution effect), or a higher reservoir species richness with a higher total abundance might increase the spillover risk. |
| Deforestation | Deforestation decreases predator species richness.^1^ | Deforestation increases contact between reservoirs and humans.^2^ |
| Population density | A high population density could indicate development, usually associated with a lower predator species richness. | The greater the population density, the greater the risk of HFRS. |
| Number of farmers | - | Farmers are at risk of HFRS.^3^ |
| Budget dependency | - | Budget dependency indicates the district-level economic status. As HFRS is prevalent in rural areas of South Korea, lower district-level economic status (rural status) may increase the HFRS risk. |
| Annual average temperature | Meteorological factors may affect predator survival. | Rodents forage more intensively at lower temperatures because more calories are required to maintain body temperature.^4^ |
| Annual precipitation | Meteorological factors may affect predator survival. | Moisture increases rodent activity and numbers.^4^ |
| Relative humidity | Meteorological factors may affect predator survival. | Moisture increases rodent activity and numbers.^4^ |
| Agricultural land use (rice field) | - | Working in rice fields is a risk factor for HFRS. |
| Urban land use | The predator species richness is lower in urban areas. | HFRS incidence is lower in rural areas. |
| Forest land use | The predator species richness is higher in forested areas. | The forested area may affect hantavirus prevalence among reservoirs.^5^ |
| Elevation | Altitude may affect wildlife species richness. | Altitude may affect human immunity.^6^ |
| Spatial extent | The larger the spatial extent, the greater the species richness. | The larger the spatial extent, the more HFRS cases. |

**Reference**

1. Betts, M. G., Wolf, C., Ripple, W. J., Phalan, B., Millers, K. A., Duarte, A., ... & Levi, T. (2017). Global forest loss disproportionately erodes biodiversity in intact landscapes. Nature, 547(7664), 441.

2. Olivero, J., Fa, J. E., Real, R., Márquez, A. L., Farfán, M. A., Vargas, J. M., ... & King, S. (2017). Recent loss of closed forests is associated with Ebola virus disease outbreaks. Scientific Reports, 7(1), 14291.

3. Zeitz, P. S., Graber, J. M., Voorhees, R. A., Kioski, C., Shands, L. A., Ksiazek, T. G., ... & Khabbaz, R. F. (1997). Assessment of occupational risk for hantavirus infection in Arizona and New Mexico. Journal of Occupational and Environmental Medicine, 39(5), 463-467.

4. Prist, P. R., Uriarte, M., Fernandes, K., & Metzger, J. P. (2017). Climate change and sugarcane expansion increase Hantavirus infection risk. PLoS neglected tropical diseases, 11(7), e0005705.

5. Goodin, D. G., Paige, R., Owen, R. D., Ghimire, K., Koch, D. E., Chu, Y. K., & Jonsson, C. B. (2009). Microhabitat characteristics of Akodon montensis, a reservoir for hantavirus, and hantaviral seroprevalence in an Atlantic forest site in eastern Paraguay. Journal of Vector Ecology, 34(1), 104-113.

6. Mishra, K. P., & Ganju, L. (2010). Influence of high altitude exposure on the immune system: a review. Immunological investigations, 39(3), 219-234.

**Supplementary material 3 – Variable selection process for avoiding multi-collinearity**

In this study, fourteen explanatory variables were acquired and 4 of them were excluded, due to high variance inflation factor (VIF) value (>10) or high correlation with other variables. (>0.7) (Table S3-1) As a result, ten explanatory variables were included in the final model.

**Table S3-1. The final list of included Variables included**

| Variables | Excluded | Reason |
| --- | --- | --- |
| Predator SR ^a^ (counts) | No | - |
| Reservoir SR ^a^ (counts) | No | - |
| Deforestation (3 years, km^2^) | No | - |
| Population density (10^3^/km^2^) | Yes | High correlation with predator SR ^a^ |
| Farmer population (counts) | Yes | High correlation with agricultural land use |
| Budget dependency (%) | No | - |
| Annual average temperature (^o^C) | No | - |
| Annual Precipitation (mm) | No | - |
| Relative humidity (%) | No | - |
| Agricultural land use (100 m^2^) | No | - |
| Urban land use (km^2^) | No | - |
| Forest land use (km^2^) | Yes | High correlation with deforestation and elevation |
| Elevation (mean, m) | No | - |
| Extent (km^2^) | Yes | High VIF ^b^ value |

^a^ SR: species richness

^b^ VIF: variance inflation factor

*Note*: Among the fourteen acquired variables, we excluded variables with high VIF value (>10) or variables which showed high correlation with other variables (correlation coefficient more than 0.7).

Variable selection process using VIF was described in Table S3-2.

**Table S3-2. Variable selection using variance inflation factor (VIF)**

| Variables | VIF ^a^ | |
| --- | --- | --- |
|  | Step 1 | Step 2 |
| Predator species richness (counts) | 4.29 | 3.54 |
| Reservoir species richness (counts) | 2.23 | 2.03 |
| Deforestation (3 years, km^2^) | 2.29 | 2.32 |
| Population density (10^3^/km^2^) | 2.45 | 2.37 |
| Farmer population (counts) | 4.24 | 3.30 |
| Budget dependency (%) | 2.24 | 2.21 |
| Annual average temperature (^o^C) | 1.47 | 1.34 |
| Annual Precipitation (mm) | 1.08 | 1.05 |
| Relative humidity (%) | 1.64 | 1.62 |
| Agricultural land use (rice paddy field, 100 m^2^) | 4.03 | 3.81 |
| Urban land use (km^2^) | 1.57 | 1.53 |
| Forest land use (km^2^) | 8.56 | 4.08 |
| Elevation (mean, m) | 3.87 | 3.54 |
| Extent (km^2^) | 15.15 | - |

^a^ VIF: variance inflation factor

*Note*: To identify variables with high VIF value, we included all explanatory variables in the model (Step 1). As the extent variable showed a VIF value higher than 10, we excluded the variable and estimated VIF values using remaining variables (Step 2).

Variable selection process using one to one correlation coefficients was illustrated in Figure S3.


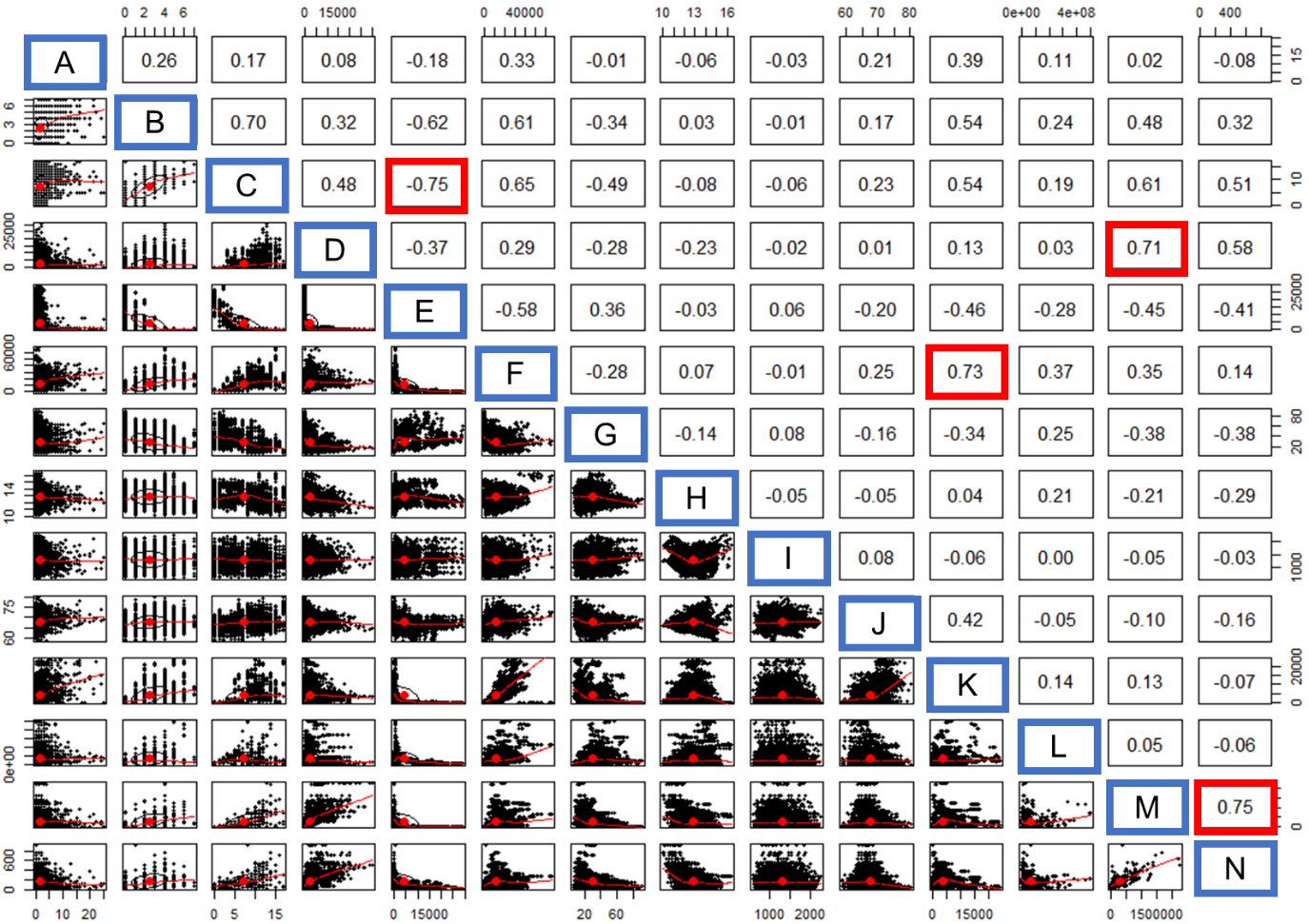

Figure S3. One to one correlation between variables

*Note*: Each capital indicates variables as follows. A= Annual number of hemorrhagic fever with renal syndrome cases, B=reservoir species richness, C=Predator species richness, D=Deforestation, E=population density, F=Farmer population, G=Budget dependency, H=Annual mean temperature, I=Annual precipitation, J=Relative humidity, K=Agricultural land use, L=Urban land use, M=Forest land use, N=elevation.

The upper triangular elements of matrix represent correlation coefficient and red box indicates the coefficient higher than 0.7. Correlation coefficient between reservoir species richness and predator species richness was 0.699, so both variables were not excluded.

Table S3-3 showed that there is no multicollinearity issue as all variables included in the final model showed GVIF^(1/(2*Df)) value lower than 2.

Table S3-3. GVIF^(1/(2*Df)) for variables included in the final model

| Variables | GVIF^(1/(2*Df)) |
| --- | --- |
| Predator species richness (Quartile) | 1.22 |
| Reservoir species richness | 1.39 |
| Deforestation (3 years) | 1.35 |
| Budget dependency (%) | 1.43 |
| Average mean temperature | 1.17 |
| Annual Precipitation | 1.02 |
| Relative humidity | 1.23 |
| Agricultural land use (rice paddy field) | 1.58 |
| Urban land use (km^2^) | 1.20 |
| Elevation (mean, m) | 1.67 |

^a^ GVIF: generalized variance inflation factor

*Note*: GVIF^(1/(2*Df)) value less than 2 indicate that there is no significant evidence for multicollinearity problem in the model

**Supplementary materials 4 – Results of association analysis for covariates**

Table S4. Associations between human hemorrhagic fever with renal syndrome incidence and covariates used in the models.

| Variables | Relative risk (95% Credible interval) | | | |
| --- | --- | --- | --- | --- |
|  | Poisson | NB | ZIP | ZINB |
| Reservoir species richness | 1.203  (1.177 – 1.229) | 1.207  (1.168 – 1.248) | 1.193  (1.166 – 1.220) | 1.207  (1.168 – 1.248) |
| Deforestation (3 year, km^2^) | 1.022  (1.005 – 1.039) | 1.032  (1.006 – 1.058) | 1.017  (0.999 – 1.035) | 1.031  (1.006 – 1.057) |
| Budget dependency (%) | 0.995  (0.992 – 0.998) | 0.996  (0.992 – 1.000) | 0.996  (0.993 – 0.999) | 0.996  (0.992 – 1.000) |
| Average mean temperature | 0.820  (0.783 – 0.859) | 0.848  (0.802 – 0.896) | 0.848  (0.808 – 0.891) | 0.848  (0.802 – 0.897) |
| Annual Precipitation  (cm) | 1.104  (0.945 – 1.291) | 0.983  (0.828 – 1.169) | 1.145  (0.975 – 1.348) | 0.984  (0.829 – 1.171) |
| Relative humidity  (%) | 1.031  (1.016 – 1.047) | 1.051  (1.032 – 1.071) | 1.030  (1.014 – 1.046) | 1.051  (1.032 - 1.071) |
| Agricultural area  (100 m^2^) | 1.005  (1.004 - 1.006) | 1.006  (1.005 – 1.008) | 1.005  (1.004 – 1.005) | 1.006  (1.005 – 1.008) |
| Urban area (km^2^) | 0.998  (0.997 – 0.998) | 0.998  (0.997 – 0.998) | 0.998  (0.997 – 0.998) | 0.998  (0.997 – 0.998) |
| Elevation (m) | 1.001  (1.000 – 1.001) | 1.000  (1.000 – 1.001) | 1.001  (1.000 – 1.001) | 1.000  (1.000 – 1.001) |
| DIC | 11569.82 | 10668.36 | 11345.20 | 10671.67 |

**Supplementary Material 5 – Wildlife species included.**

We included 10 known reservoirs of hantavirus in South Korea: the striped field mouse^1^, Korean field mouse^2^, grey red-backed vole^3^, Disinezumi shrew^4^, Ussuri white-toothed shrew^5^, Asian lesser white-toothed shrew^6^, Eurasian harvest mouse^5^, reed vole^5^, brown rat,^7^ and Southern red-backed vole (Table S5-1).

**Table S5-1. Hantavirus reservoirs.**

| Academic name | IUCN common name |
| --- | --- |
| *Apodemus agrarius* | Striped field mouse |
| *Apodemus pennisulae* | Korean field mouse |
| *Clethrionomys rufocanus* | Grey red-backed vole |
| *Crocidura dsinezumi* | Dsinezumi shrew |
| *Crocidura lasiura* | Ussuri white-toothed shrew |
| *Crocidura shantungensis* | Asian lesser white-toothed shrew |
| *Micromys minutus* | Eurasian harvest mouse |
| *Microtus fortis* | Reed vole |
| *Rattus norvegicus* | Brown rat |
| *Myodes regulus* | Southern red-backed vole |

In terms of mammals, we included seven predator species, listed below (Table S5-2).

**Table S5-2. Mammalian predator species.**

| Academic name | IUCN Common name |
| --- | --- |
| *Meles leucurus* | Asian badger |
| *Mustela sibirica* | Siberian weasel |
| *Mustela nivalis* | Least weasel |
| *Martes flavigula* | Yellow-throated marten |
| *Lutra lutra* | Eurasian otter |
| *Nyctereutes procyonoides* | Raccoon dog |
| *Prionailurus bengalensis* | Leopard cat |

In terms of avian predators, we included the 24 predator species below (Table S5-3).

**Table S5-3. Avian predator species included (Falconidae, Strigidae)**

| Academic name | IUCN common name |
| --- | --- |
| *Accipiter gularis* | Japanese sparrowhawk |
| *Accipiter nisus* | Eurasian sparrowhawk |
| *Accipiter gentilis* | Northern goshawk |
| *Butastur indicus* | Grey-faced buzzard |
| *Buteo buteo* | Eurasian buzzard |
| *Buteo hemilasius* | Upland buzzard |
| *Buteo lagopus* | Rough-legged buzzard |
| *Aquila chrysaetos* | Golden eagle |
| *Accipiter soloensis* | Chinese sparrowhawk |
| *Circus melanoleucos* | Pied harrier |
| *Milvus migrans* | Black kite |
| *Haliaeetus albicilla* | White-tailed sea eagle |
| *Haliaeetus pelagicus pelagicus* | Steller's sea eagle |
| *Circus spilonotus* | Eastern marsh-harrier |
| *Circus cyaneus* | Hen harrier |
| *Falco tinnunculus* | Common kestrel |
| *Falco amurensis* | Amur falcon |
| *Falco columbarius* | Merlin |
| *Falco peregrinus* | Peregrine falcon |
| *Athene noctua* | Little owl |
| *Asio flammeus* | Short-eared owl |
| *Strix aluco* | Tawny owl |
| *Bubo bubo* | Eurasian eagle-owl |
| *Otus bakkamoena* | Indian scops owl |

**Reference**

1. Baek, L. J., Kim, K. S., Song, K. J., Kho, E. Y., Chung, K. M., Park, K. S., ... & Song, J. W. (1999). Seroepidemiological study on hantavirus infection of wild rodents captured in the moutainous areas of Korea. The Journal of Korean Society of Virology, 29(1), 1-9.

2. Song, K. J., Yun, H. S., Kho, E. Y., Chung, K. M., Park, K. S., Lee, Y. J., ... & Baek, L. J. (2000). Isolation of Apodemus peninsulae-borne Hantavirus and Comparison of Molecular Biological Characteristics. The Journal of Korean Society of Virology, 30(1), 19-28.

3. Song, H. J., Lee, D. Y., Kim, C. M., & Shin, Y. H. (2006). Epidemiological Survey of Hantaan Virus Infection of Wild Rodents Trapped in Jeollanam-do, 2003~ 2004. Journal of Bacteriology and Virology, 36(3), 205-210.

4. Song, J. W., Kang, H. J., Gu, S. H., Moon, S. S., Bennett, S. N., Song, K. J., ... & Klein, T. A. (2009). Characterization of Imjin virus, a newly isolated hantavirus from the Ussuri white-toothed shrew (Crocidura lasiura). Journal of virology, 83(12), 6184-6191.

5. Kim, H. C., Kim, W. K., Klein, T. A., Chong, S. T., Nunn, P. V., Kim, J. A., ... & Song, J. W. (2017). Hantavirus surveillance and genetic diversity targeting small mammals at Camp Humphreys, a US military installation and new expansion site, Republic of Korea. PloS one, 12(4).

6. Arai, S., Gu, S. H., Baek, L. J., Tabara, K., Bennett, S. N., Oh, H. S., ... & Okabe, N. (2012). Divergent ancestral lineages of newfound hantaviruses harbored by phylogenetically related crocidurine shrew species in Korea. Virology, 424(2), 99-105.

7. Kim, Y. S., Ahn, C., Han, J. S., Kim, S., Lee, J. S., & Lee, P. W. (1995). Hemorrhagic fever with renal syndrome caused by the Seoul virus. Nephron, 71(4), 419-427.

8. Song, K. J., Baek, L. J., Moon, S., Ha, S. J., Kim, S. H., Park, K. S., ... & Yanagihara, R. (2007). Muju virus, a novel hantavirus harboured by the arvicolid rodent Myodes regulus in Korea. The Journal of general virology, 88(Pt 11), 3121.

**Supplementary Material 6 – The results of association analyses using generalized additive models.**

| **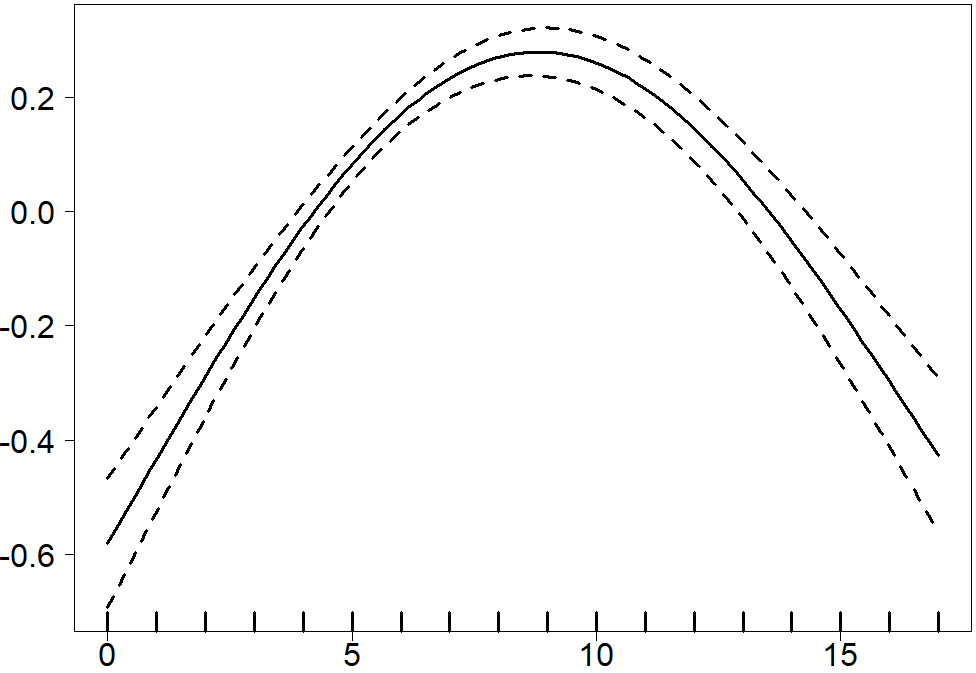** | **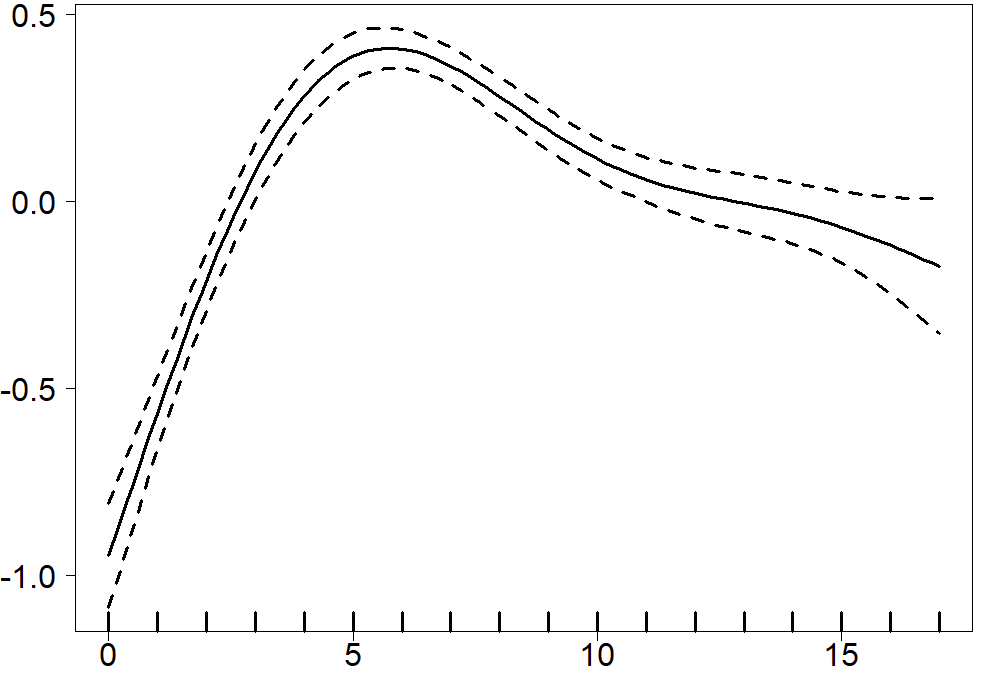** |
| --- | --- |
| **k=3, AIC=12166.62** | **k=5, AIC=12038.8** |
| **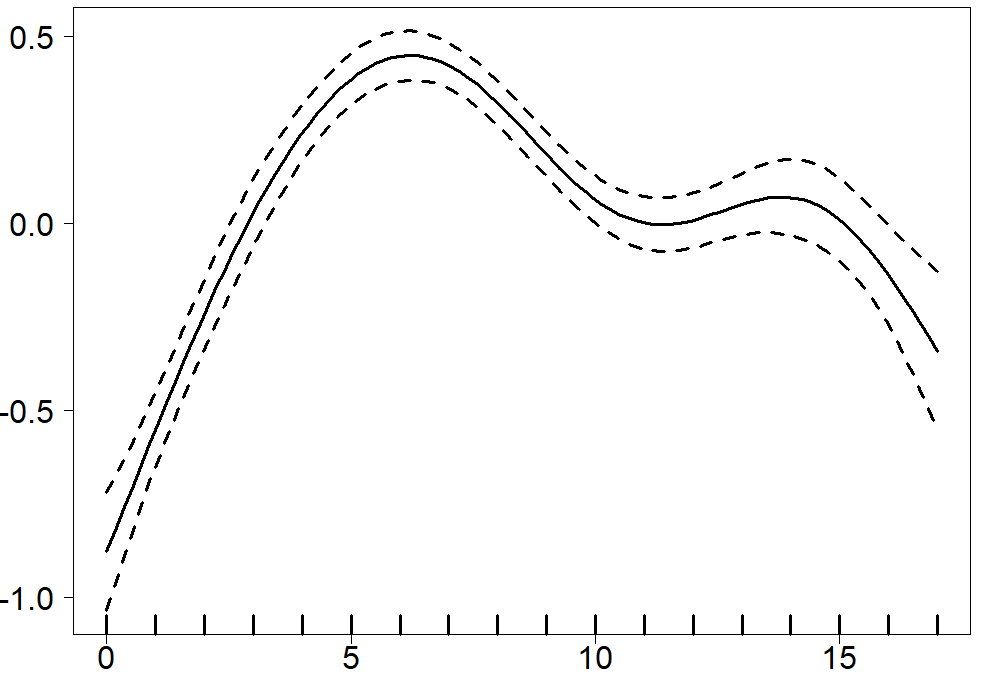** | **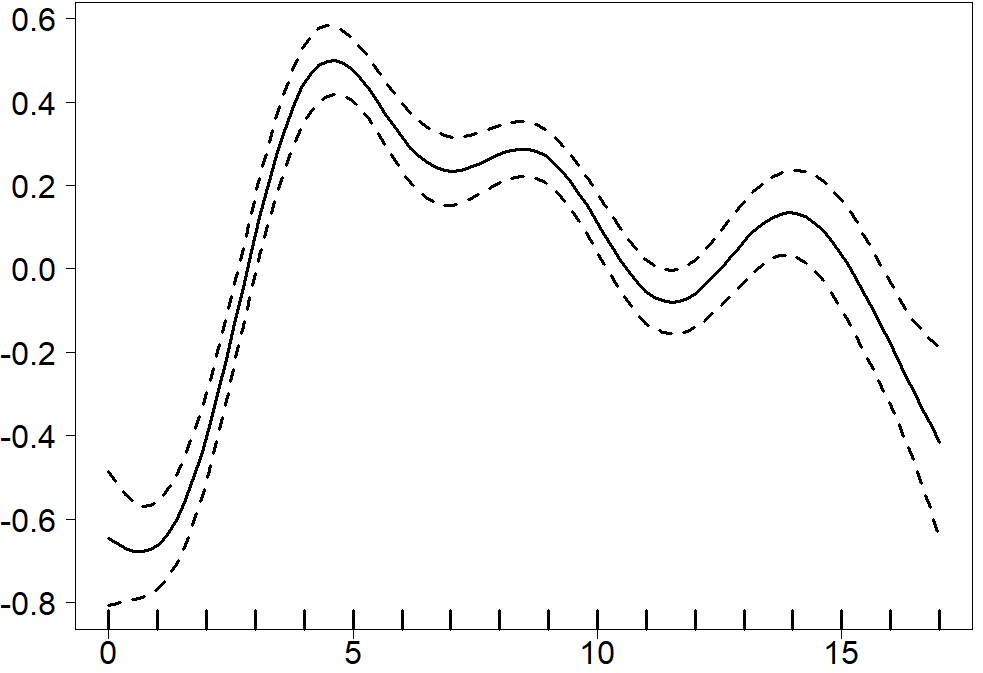** |
| **k=7, AIC=12030.4** | **k=9, AIC=11980.68** |

Figure S6. We used generalized additive models for preliminary analyses of possible non-linear associations between predator species richness and the incidence of haemorrhagic fever with renal syndrome (HFRS) in South Korea

*Note*: The Poisson distribution was employed; the outcome variable and main explanatory variable were the number of HFRS cases and predator species richness, respectively. The covariates were reservoir species richness, extent of deforestation, budget dependency, annual mean temperature, annual precipitation, relative humidity, agricultural area, urban area, and elevation. x-axis: predator species richness; y-axis: log relative risk.
